# Canine Multiple System Degeneration Associated with Sequence Variants in *SERAC1*

**DOI:** 10.3390/genes15111378

**Published:** 2024-10-25

**Authors:** Rong Zeng, Juyan Guo, Garrett Bullock, Gary S. Johnson, Martin L. Katz

**Affiliations:** 1 IWK Health, Pathology and Laboratory Medicine, 5850 University Avenue, Halifax, NS B3K 6R8, Canada; zengtony743@gmail.com; 2 Canine Genetics Laboratory, Department of Veterinary Pathobiology, College of Veterinary Medicine, University of Missouri, Columbia, MO 65211, USA; guoj@missouri.edu (J.G.); gebkd2@missouri.edu (G.B.);; 3 Neurodegenerative Diseases Research Laboratory, Department of Ophthalmology, University of Missouri, Columbia, MO 65212, USA

**Keywords:** movement disorder, dog, mitochondria, whole-genome sequencing, neurodegeneration

## Abstract

Canine multiple system degeneration (CMSD) is an early onset, progressive movement disorder affecting Kerry Blue Terriers and Chinese Crested dogs. The associated pathologic lesions include degeneration of the cerebellum, caudate nucleus, and substantia nigra. CMSD is inherited as an autosomal recessive trait in both dog breeds. Previous linkage mapping localized the CMSD locus to a 15 MB region on canine chromosome 1 (CFA1). Next-generation sequencing was used to generate whole-genome sequences from the DNA of an affected dog from each breed. The resulting sequence reads were aligned to the NCBI canine reference genome (build 3.1). Among the homozygous sequence variants within the CFA1 target region, a nonsense variant in exon 15 of *SERAC1* was identified in the affected Kerry Blue Terrier, while in the Chinese Crested dog, a 4 bp deletion in the *SERAC1* exon 4 acceptor splice site was found. RT-PCR showed that this deletion resulted in exon 4 skipping. Genotyping of large cohorts of Kerry Blue Terriers and Chinese Crested dogs for the respective breed-specific *SERAC1* variants showed complete concordance between genotype and disease phenotype. Genotype–phenotype concordance was also observed in offspring generated by cross breeding between *SERAC1*-heterozygous Kerry Blue Terrier and Chinese Crested dogs, with only the compound heterozygotes exhibiting the disease phenotype, further confirming the recessive inheritance of CMSD. Variants in human *SERAC1* are associated with disorders with a range of ages of disease onset and patterns of clinical signs, but that are all characterized by movement abnormalities similar to those of the dogs with CMSD. Canine CMSD could serve as a valuable model to elucidate the mechanisms underlying SERAC1-deficiency disorders and to evaluate potential therapeutic interventions.

## 1. Introduction

Canine multiple system degeneration (CMSD) is a progressive early onset movement disorder that occurs in Kerry Blue Terrier and Chinese Crested dogs [1,2]. Disease onset is characterized by cranial intention tremor and cerebellar ataxia that first become apparent at 3 to 6 months of age. The affected dogs exhibit a goose-stepping gait and infrequent falls during this early stage. By 6 to 8 months of age, the falling episodes become more frequent and the gait changes to festination with dysmetria. As the disease progresses, the affected dogs develop akinesia and severe postural instability and frequent falls. Magnetic resonance imaging of the brains demonstrated disease-related cerebellar atrophy. Due to the progressive severity of neurologic signs, affected dogs are typically euthanized humanely by 13 to 18 months of age. Postmortem examination of the brains revealed Purkinje cell loss in the cerebellum and neuronal loss from the substantia nigra, putamen, and caudate nucleus [1]. The patterns of inheritance in both breeds indicate that CMSD is an autosomal recessive disorder.

Previous microsatellite-based linkage mapping localized the CMSD disease loci to chromosome 1 in both breeds [1]. Further haplotype analysis using 11 additional microsatellite markers on CFA1 narrowed the target region to a 15 MB region containing 89 positional candidate genes. Among the genes in this region is *PARK2*, a gene associated with autosomal recessive juvenile parkinsonism, which presents with similar signs to CMSD including akinesia, gait abnormalities, and postural instability [3]. Degeneration of neurons in the substantia nigra, loss of Purkinje cells, and cerebellar ataxia are also reported in some human patients with Parkinson-plus syndromes [4,5,6,7]. Given these similarities, it was hypothesized that variants in *PARK2* may be responsible for CMSD. However, this has not yet been directly tested. To identify the underlying genetic variants underlying CMSD, whole-genome sequence analyses were performed on affected dogs from both Kerry Blue Terrier and Chinese Crested breeds.

## 2. Materials and Methods

### 2.1. Interbreed Mating Study

Because the disease phenotypes in the Chinese Crested and Kerry Blue Terrier dogs were identical and the causal variants were mapped to the same region of CFA1 in both breeds, we hypothesized that the disease in both breeds resulted from variants in the same gene. To test this hypothesis, we evaluated the pattern of inheritance of the disease phenotype in offspring by crossing obligate carriers from each breed (Chinese Crested X Kerry Blue Terrier). The offspring of this cross were monitored for the development of the characteristic CMSD phenotype. This breeding experiment was approved by the University of Missouri Institutional Review Board (protocol 20520, approved 22 December 2011).

### 2.2. Whole-Genome Sequence Analyses

Genomic DNA was isolated from blood leukocytes from an affected Kerry Blue Terrier and an affected Chinese Crested dog, as described previously [8]. DNA samples were submitted to the University of Missouri Genomics Technology Core Facility for library construction and 2 × 150-base pair paired-end sequencing on an Illumina HiSeq 2000, with a target coverage of 20×. Raw sequence reads were aligned to the canine reference genome (build 3.1) by a modified Burrows–Wheeler Transform (BMT) alignment method using NextGENe v2.3.3 software, which takes into account parameters including mapping quality, Phred quality for each base, read depth, repetitiveness, and minor allele frequency. Sequence variant calling and reference sequence annotation were performed by integrating the NCBI canine genome reference sequence annotation with NextGENe software.

To identify candidate pathogenic mutations, we generated a sequence variant report table for each dog that listed all the homozygous variants detected in the coding sequences and flanking intronic sequences relative to the reference genome. This included homozygous splice site variants, nonsynonymous missense variants, indel variants, stop to read-through variants, and premature stop codon variants. The sequence variants were further filtered to exclude homozygous variants that were present in the whole-genome sequences of 102 dogs that had not exhibited signs of movement disorders. These whole-genome sequences had been generated previously by us using approaches similar to those used in this study. By focusing only on the sequence variants within the CFA1 disease target region identified by our previous linkage analysis, we generated final variant reports for each of the CMSD-affected dogs.

Among these sequence variants were a G to A substitution in *SERAC1* in the affected Kerry Blue Terrier and a 4 bp deletion in *SERAC1* in the Chinese Crested dog. Individual canine DNA samples were genotyped with respect to the 4 bp deletion by direct re-sequencing the DNA using an automated Sanger sequencer (3730 xl; Applied Biosystems, Waltham, MA, USA). The forward primer was 5′-GGAAATATAATAAAGTTTACTGG-3′ and reverse primer was 5′- CAAAATTTATACATATTTGCCAC-3′. The resulting amplicon was 140 bp in length. Individual canine DNA samples were genotyped by RFLP-PCR with respect to the G to A nucleotide change, which produced a premature stop codon. The PCR forward primer was 5′- CCCAATAAAAGCTCTTGCCT-3′ and reverse primer was 5′- GGCCAGAATTAAGTGAACCA-3′. The restriction enzyme BstXI was used to digest the PCR product using the following reaction conditions: 3U BstXI enzyme and 1X NEBuffer 3, incubated at 37 °C for 2 h. For the ancestral allele, the 299 bp PCR product remains intact, whereas the digestion cuts the mutant allele into 182 bp and 117 bp oligonucleotides. Restriction fragment sizes were determined with a microcapillary electrophoresis system (QIAxel, Qiagen, Venlo, The Netherlands).

TRIzol Reagent (Invitrogen) was used to extract the total RNA from the brains of one affected and two unaffected Chinese Crested dogs. The affected dog was euthanized due to the progression of the movement disorder signs and the unaffected dogs were euthanized due to unrelated health issues. RT-PCR amplifications were performed with a GeneAmp^®^EZ *rTth* RNA PCR kit (Applied Biosystems) using the following primer pair: 5′-TGCAGAAGAATAGGAACCTCA-3′ and 3′-TTGCCTGGTAGGTGATTCCAT-5′. The resulting amplicons were evaluated with a microcapillary electrophoresis system (QIAxcel, Qiagen, Venlo, The Netherlands).

## 3. Results

**Figure 1:**
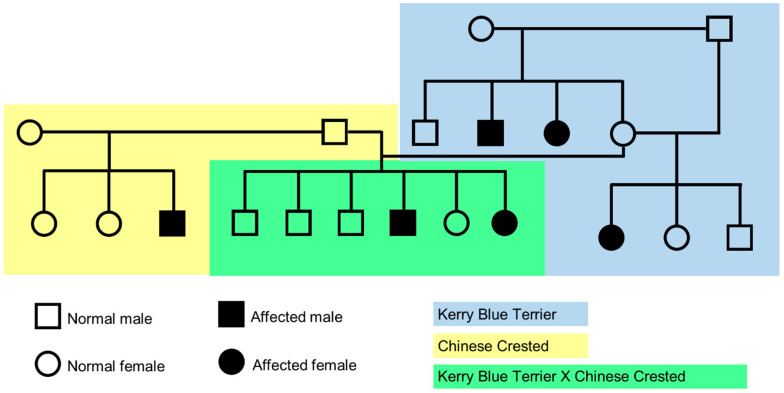
Pedigree showing segregation consistent with an autosomal recessive inheritance pattern for CMSD and that the causal variants likely occurred in the same gene in the two breeds. The background is blue behind the Kerry Blue Terrier family, yellow behind the Chinese Crested dogs, and green behind the cross-bred litter. (Adapted from [1].)

Litters of both Kerry Blue Terriers and Chinese Crested dogs from unaffected parents included both CMSD-affected and unaffected offspring (Figure 1), indicating that the disorder is a recessive trait in both breeds. The cross breeding of obligate carrier Kerry Blue Terrier and Chinese Crested dogs produced a litter that included both CMSD-affected and unaffected offspring (Figure 1). This finding indicated that the casual variants likely reside in the same gene in the two breeds.

The whole-genome sequence from a CMSD-affected Chinese Crested dog consisted of 167,497,918 reads, of which, 120,222 were duplicate reads and 167,377,696 were unique. The whole-genome sequence from an affected Kerry Blue Terrier consisted of 384,505,652 reads, of which, only 360 were duplicate reads and 384,505,292 were unique. The unique reads generated from both dogs were aligned separately to the canine reference genome using NextGENe software, resulting in an aligned sequence with an average of 18-fold coverage for the Chinese Crested dog and an average of 22.3-fold coverage for the Kerry Blue Terrier. For each dog, the sequence variants were filtered to include only those that were homozygous in the probands. Variants found to be homozygous in the whole-genome sequences of any of the 102 other dogs in our internal database that did not suffer from CMSD were also excluded. The remaining homozygous variants were further filtered to include only those located within the CFA1 region to which we previously mapped the causal variant [1].

**Figure 2:**
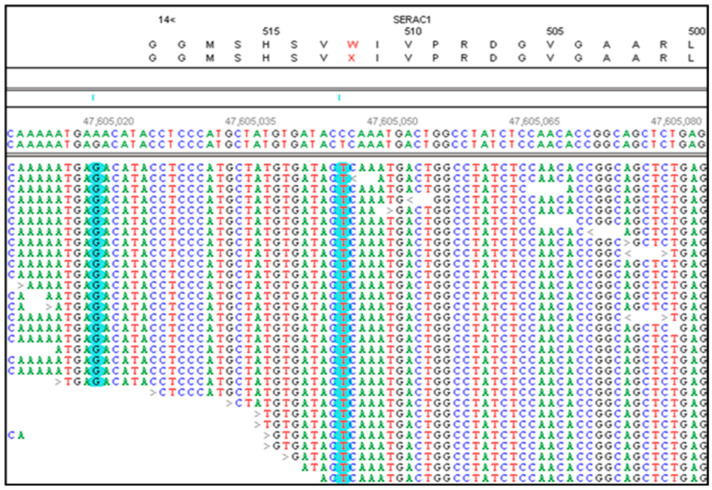
NextGENe software viewer shows an alignment of whole-genome sequence reads of DNA from a CMSD-affected Kerry Blue Terrier in the antisense orientation for *SERAC1*. This region contains a homozygous C > T which converts a tyrosine codon (TGG on the sense strand) to a premature stop codon (TGA on the sense strand). Sequence variants are highlighted in blue.

Two variants in the Kerry Blue Terrier met the filtering criteria: a nonsense variant in *SERAC1* (Figure 2) and a missense variant in *SLC22A2*. The *SERAC1* variant, which introduces a premature stop codon (p. W512X) in exon 15, was considered more likely to be causal due to its predicted impact on the encoded protein and the associations of variants in this gene with neurological disorders [9].

**Figure 3:**
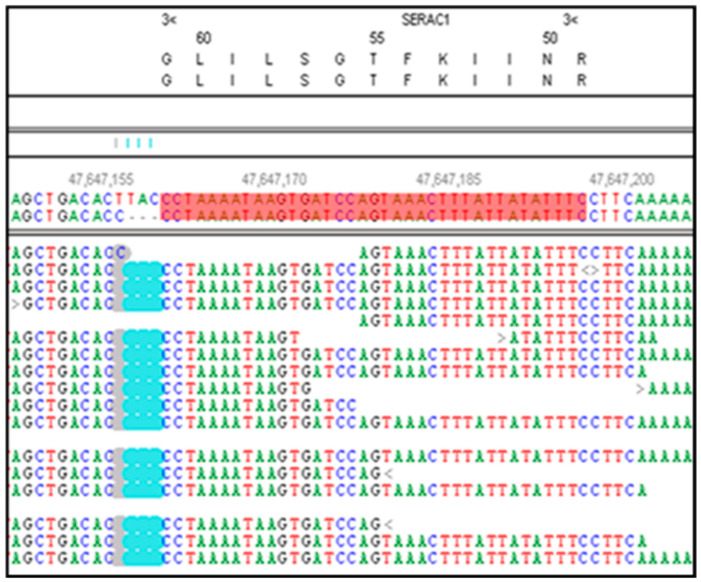
NextGENe software viewer shows an alignment of whole-genome sequence reads generated from the DNA from a CMSD-affected Chinese Crested dog in the antisense orientation for *SERAC1*. This region contains a homozygous 4 bp deletion (GTAA in the antisense strand) in the exon 4 acceptor splice site. The deleted nucleotides are highlighted in blue. The *SERAC1* exon 4 coding region is highlighted in red.

**Figure 4:**
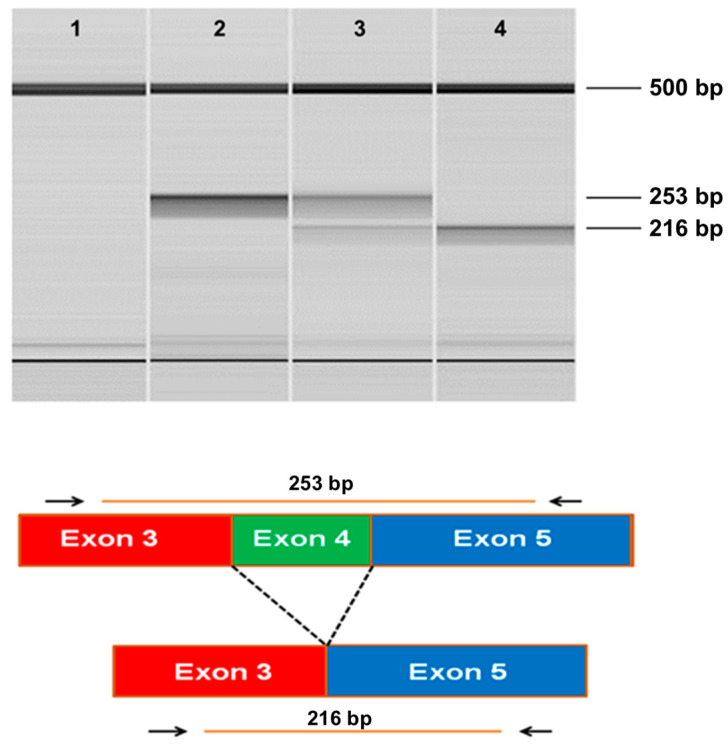
Microcapillary electrophoretograms of RT-PCR amplicons produced with primers designed to anneal to exon 3 and exon 5 of *SERAC1*. The RNA from a CMSD-affected (lane 4) and two unaffected (lanes 2 and 3) Chinese Crested dogs. A band at 253 bp was expected from the consecutive splicing of exon 3 to exon 4 to exon 5. A band at 216 bp was expected from the splicing of exon 3 to exon 5. Lane 1 is a no-template control (lane 1). The band at 500 bp is from an internal molecular weight marker.

**Figure 5:**
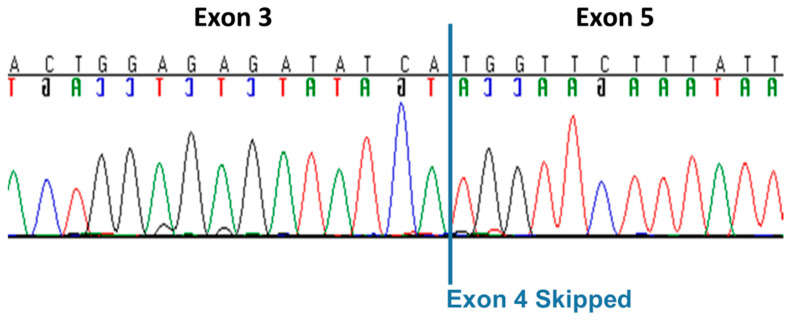
Automated Sanger sequence electrophoretograms of the RT-PCR amplicon produced with RNA from a CMSD-affected Chinese Crested dog confirming the splicing of *SERAC1* exon 3 to exon 5.

The whole-genome sequence of the CMSD-affected Chinese Crested dog contained a 4 bp (GTAA) deletion in the acceptor splice site of *SERAC1* exon 4 (Figure 3). SplicePort (http://spliceport.cbcb.umd.edu/SplicingAnalyser.html, accessed on 5 February 2013), an interactive splice site analysis tool, predicted that the mutation would cause exon skipping. This prediction was confirmed by the RT-PCR amplification of *SERAC1* transcripts between exon 3 and exon 5, which showed a reduced transcript length in RNA from an affected Chinese Crested dog corresponding to the omission of exon 4 (Figure 4). Automated Sanger sequencing of the RT-PCR product confirmed exon 4 skipping in the transcript from the affected dog (Figure 5).

**Figure 6:**
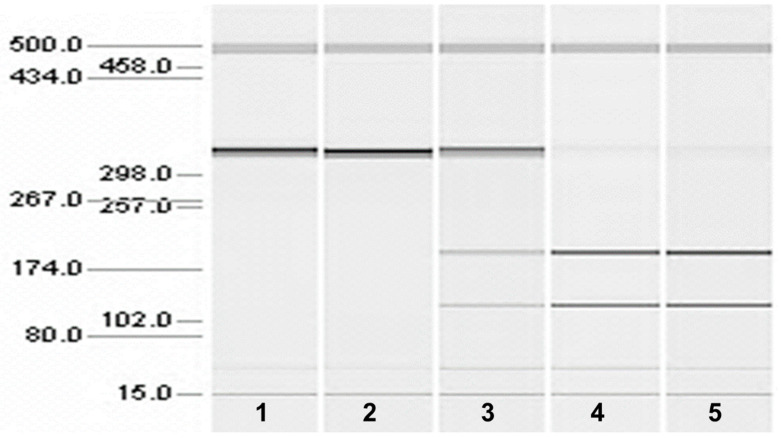
Microcapillary electrophoretograms of amplicons produced from the RFLP-PCR genotyping assay with DNA from two CMSD-affected KBTs (lanes 1 and 2), DNA from a clinically normal KBT with a “carrier” (heterozygous) test result (lane 3), and two other clinically normal KBTs with a homozygous normal test result (lanes 4 and 5). The bands at 15 bp and 500 bp are from internal molecular weight markers.

A PCR-RFLP assay was used to genotype individual Kerry Blue Terriers for the *SERAC1* nonsense variant (Figure 6). Genotyping was performed on a cohort of 228 Kerry Blue Terriers with known clinical statuses. All CMSD-affected dogs were homozygous for the variant allele, while clinically unaffected dogs were either homozygous for the reference allele or heterozygous. Homozygosity for the mutant allele was highly significantly associated with the CMSD phenotype (*p* < 1.0 × 10^−9^, Fisher’s exact test 2 × 2). Table 1 summarizes the genotype distribution. Ninety dogs from 25 other breeds that did not exhibit CMSD signs were all homozygous for the reference allele.

**Figure 7:**
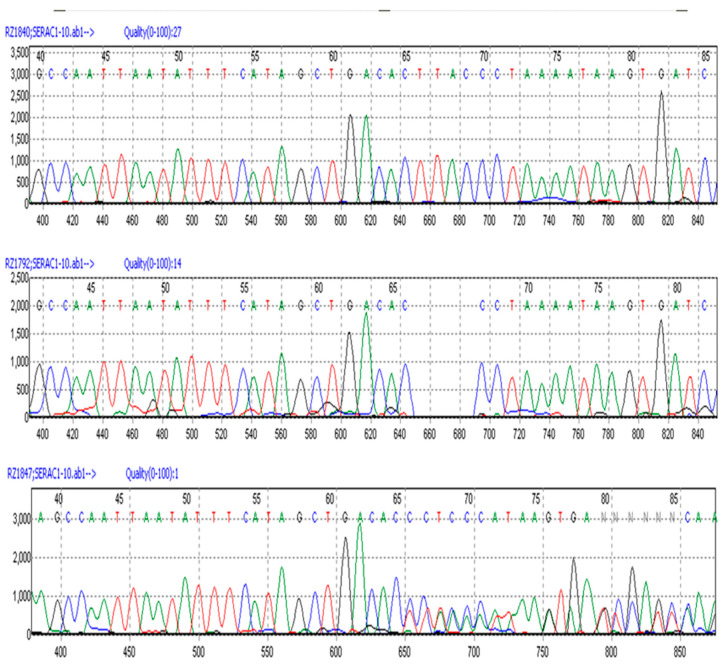
Automated Sanger sequence electrophoretograms of PCR amplicons produced from DNA from unaffected (top), CMSD-affected (middle), and heterozygous unaffected Chinese Crested dogs (bottom).

A cohort of 183 Chinese Crested dogs with known clinical statuses was genotyped to screen for the *SERAC1* 4 bp deletion using automated Sanger sequencing of PCR amplicons generated with primers spanning the deletion site (Figure 7). Table 2 summarizes the genotype and phenotype distributions. All 41 CMSD-affected dogs were homozygous for the mutant allele, while clinically unaffected dogs were either homozygous for the reference allele or heterozygous. Homozygosity for the mutant allele was highly significantly associated with the CMSD phenotype (*p* < 1.0 × 10^−21^, Fisher’s exact test 2 × 2). Of the 131 dogs from 11 other breeds that did not exhibit CMSD signs, all were homozygous for the reference allele.

## 4. Discussion

Hereditary CMSD was described in Kerry Blue Terriers as early as 1946 [2,10] and subsequently in Chinese Crested dogs [1]. Linkage analyses using microsatellite markers localized the genetic variants underlying the disease to a 15 Mb region of CFA1 in both breeds, but the specific variants underlying the disorder in these breeds were not identified. Because the clinical signs of CMSD resemble those in some forms of human Parkinson’s disease associated with variants in *PARK2*, and because the canine ortholog of *PARK2* is located within the mapped CFA1 region, it was hypothesized that variants in *PARK2* might underlie CMSD in both breeds. However, the whole-genome sequences of CMSD-affected dogs from both breeds did not contain homozygous variants in *PARK2* relative to the reference sequence.

With advances in technology and improved annotation of the canine genome, we were able to identify the causal variants in *SERAC1* in both breeds. Canine *SERAC1* encodes two protein isoforms that are 672 and 654 amino acids in size. The larger isoform has 18 more amino acids at the N-terminus that are encoded by an exon that is not utilized for the shorter isoform. Other than that, the amino acid sequences of both isoforms are identical. The *SERAC1* gene includes 17 exons with the shorter isoform utilizing only exons 2–17. The *SERAC1* variant in Kerry Blue Terriers with CMSD predicts protein isoforms that would be truncated by 160 amino acids at the carboxy terminus and would therefore be unlikely to retain biological function. The *SERAC1* variant in Chinese Crested dogs with CMSD results in a transcript that is missing exon 4 and results in a frameshift starting at the exons 3–5 junction that would preclude the synthesis of functional SERAC1 protein. Thus, both variants can be considered nulls.

The SEARAC1 protein is localized to the outer mitochondrial membrane where it facilitates serine transport from the cytosol to the mitochondria [11]. SERAC1 has also been shown to play a role in modulating mitochondrial membrane phospholipid composition and intracellular cholesterol trafficking [12]. The lack of functional SERAC1 protein results in a depletion of mitochondrial DNA, leading to mitochondrial dysfunction [11]. Thus, it appears likely that the signs of CMSD are the result of impaired mitochondrial function. The evidence suggests that the mitochondrial dysfunction resulting from SERAC1 deficiency is due at least in part to an insufficient supply of nucleotides to the mitochondria, and that supplementation with nucleosides or nucleotides can at least partially restore mitochondrial function [11].

Variants in human *SERAC1* are associated with a spectrum of disorders, including MEGDHEL syndrome (3-methylglutaconic aciduria with deafness–dystonia, hepatopathy, encephalopathy, and Leigh-like syndrome) [9,13,14,15], juvenile-onset complicated spastic paraplegia [16], and adult-onset generalized dystonia [9,17]. The age of onset and the spectrum and severity of signs in human *SERAC1*-associated disorders varies widely. MEGDHEL, the most severe form, typically presents with neonatal or infantile onset with signs that include transient hypoglycemia, feeding problems, failure to thrive, optic atrophy, developmental delay followed by motor and cognitive regression, progressive sensorineural hearing loss, progressive dystonia, and progressive spasticity [9,14,18]. Most children with this form of SERAC1 deficiency are completely dependent on care for all activities of daily living, and in some cases, the disease is fatal. Over 40 different *SERAC1* variants, including missense, splice site, frame shift, and nonsense variants, have been found to be associated with the early-onset MEGDHEL [13,14,15,19,20]. A juvenile-onset disorder associated with SERAC1 deficiency is characterized by cognitive delay and slowly progressive lower limb spasticity beginning in adolescence [16]. This disorder was associated with a splice variant that resulted in the absence of full-length SERAC1 protein. The adult-onset disease associated with SERAC1 deficiency is characterized by cognitive regression and progressive dystonia beginning in early adulthood [17,21,22].

All of the human SERAC1 deficiency disorders are characterized by dystonia, which is the most prominent sign in the canine disease. Although cognitive function and hearing loss were not assessed in the affected dogs, the canine disorder appears to closely resemble early-onset MEGDHEL syndrome. Because dogs with SERAC1-related CMSD could serve as a valuable model to test therapeutic interventions for the human disorders, further phenotypic characterization of the canine disease is warranted. Evidence suggests that nucleotide supplementation corrects the SERAC1 deficiency-related mitochondrial dysfunction and therefore may have therapeutic benefits [11]. This could be tested in dogs with CMSD.

## Figures and Tables

**Table 1:** Distribution of genotypes and phenotypes among affected and unaffected Kerry Blue Terriers.

Phenotype	Genotype			Total
	Mut/Mut	Mut/WT	WT/WT	
Affected	5	0	0	5
Unaffected	0	11	212	223
Total	5	11	212	228

**Table 2:** Distribution of genotypes and phenotypes among affected and unaffected Chinese Crested dogs.

Phenotype	Genotype			Total
	Mut/Mut	Mut/WT	WT/WT	
Affected	41	0	0	41
Unaffected	0	55	87	142
Total	41	55	87	183

## Data Availability

DNA sequence data for the dogs included in this study have been archived and deposited in the NCBI Sequence Read Archive as BioSamples SAMN03580379 and SAMN03580387.

## References

[1] O’Brien D.P., Johnson G.S., Schnabel R.D., Khan S., Coates J.R., Johnson G.C., Taylor J.F. (2005). Genetic Mapping of Canine Multiple System Degeneration and Ectodermal Dysplasia Loci. J. Hered..

[2] deLahunta A., Averill D.R.J. (1976). Hereditary Cerebellar Cortical and Extrapyramidal Nuclear Abiotrophy in Kerry Blue Terriers. J. Am. Vet. Med. Assoc..

[3] Shimura H., Hattori N., Kubo S., Mizuno Y., Asakawa S., Minoshima S., Shimizu N., Iwai K., Chiba T., Tanaka K. (2000). Familial Parkinson Disease Gene Product, Parkin, Is a Ubiquitin-Protein Ligase. Nat. Genet..

[4] Shin H.-W., Hong S.-W., Youn Y.C. (2022). Clinical Aspects of the Differential Diagnosis of Parkinson’s Disease and Parkinsonism. J. Clin. Neurol..

[5] Armstrong M.J., McFarland N. (2019). Recognizing and Treating Atypical Parkinson Disorders. Handb. Clin. Neurol..

[6] Glasmacher S.A., Leigh P.N., Saha R.A. (2017). Predictors of Survival in Progressive Supranuclear Palsy and Multiple System Atrophy: A Systematic Review and Meta-Analysis. J. Neurol. Neurosurg. Psychiatry.

[7] Keir G., Roytman M., Mashriqi F., Shahsavarani S., Franceschi A.M. Atypical Parkinsonian Syndromes: Structural, Functional, and Molecular Imaging Features. AJNR Am. J. Neuroradiol..

[8] Katz M.L., Khan S., Awano T., Shahid S.A., Siakotos A.N., Johnson G.S. (2005). A Mutation in the CLN8 Gene in English Setter Dogs with Neuronal Ceroid-Lipofuscinosis. Biochem. Biophys. Res. Commun..

[9] Wortmann S.B., de Brouwer A.P.M., Wevers R.A., Morava E. (1993). SERAC1 Deficiency.

[10] Metler F., Goss L. (1946). Canine Chorea Due to Striatocerebellar Degeneration of Unknown Etiology. J. Am. Vet. Med. Assoc..

[11] Fang H., Xie A., Du M., Li X., Yang K., Fu Y., Yuan X., Fan R., Yu W., Zhou Z. (2022). SERAC1 Is a Component of the Mitochondrial Serine Transporter Complex Required for the Maintenance of Mitochondrial DNA. Sci. Transl. Med..

[12] Wortmann S.B., Vaz F.M., Gardeitchik T., Vissers L.E.L.M., Renkema G.H., Schuurs-Hoeijmakers J.H.M., Kulik W., Lammens M., Christin C., Kluijtmans L.A.J. (2012). Mutations in the Phospholipid Remodeling Gene SERAC1 Impair Mitochondrial Function and Intracellular Cholesterol Trafficking and Cause Dystonia and Deafness. Nat. Genet..

[13] Unal O., Ozgul R.K., Yucel D., Yalnizoglu D., Tokatli A., Sivri H.S., Hismi B., Coskun T., Dursun A. (2015). Two Turkish Siblings with MEGDEL Syndrome Due to Novel SERAC1 Gene Mutation. Turk. J. Pediatr..

[14] Snanoudj S., Mordel P., Dupas Q., Schanen C., Arion A., Gerard M., Read M.-H., Nait Rabah D., Goux D., Chapon F. (2019). Identification of a Novel Splice Site Mutation in the SERAC1 Gene Responsible for the MEGDHEL Syndrome. Mol. Genet. Genomic Med..

[15] Alagoz M., Kherad N., Turkmen S., Bulut H., Yuksel A. (2020). A Novel Mutation in the SERAC1 Gene Correlates with the Severe Manifestation of the MEGDEL Phenotype, as Revealed by Whole-Exome Sequencing. Exp. Ther. Med..

[16] Roeben B., Schule R., Ruf S., Bender B., Alhaddad B., Benkert T., Meitinger T., Reich S., Bohringer J., Langhans C.-D. (2018). SERAC1 Deficiency Causes Complicated HSP: Evidence from a Novel Splice Mutation in a Large Family. J. Med. Genet..

[17] Martins E., Duraes J., Nogueira C., Gomes J., Vilarinho L., Macario C. (2023). SERAC1 Deficiency- A New Phenotype. Endocr. Metab. Immune Disord. Drug Targets.

[18] Finsterer J., Scorza F.A., Fiorini A.C., Scorza C.A. (2020). MEGDEL Syndrome. Pediatr. Neurol..

[19] Maas R.R., Iwanicka-Pronicka K., Kalkan Ucar S., Alhaddad B., AlSayed M., Al-Owain M.A., Al-Zaidan H.I., Balasubramaniam S., Baric I., Bubshait D.K. (2017). Progressive Deafness-Dystonia Due to SERAC1 Mutations: A Study of 67 Cases. Ann. Neurol..

[20] Radha Rama Devi A., Lingappa L. (2018). Novel Mutations in SERAC1 Gene in Two Indian Patients Presenting with Dystonia and Intellectual Disability. Eur. J. Med. Genet..

[21] Giron C., Roze E., Degos B., Meneret A., Jardel C., Lannuzel A., Mochel F. (2018). Adult-Onset Generalized Dystonia as the Main Manifestation of MEGDEL Syndrome. Tremor Other Hyperkinet Mov..

[22] Ashton C., Davis M., Laing N., Ravenscroft G., Lamont P. (2023). Novel SERAC1 Variant Presenting with Adult-Onset Extrapyramidal Dystonia-Parkinsonism Phenotype: A Case Report. Neurol. Genet..

